# Widespread occurrence of ‘*Candidatus* Phytoplasma ulmi’ in elm species in Germany

**DOI:** 10.1186/s12866-020-01749-z

**Published:** 2020-03-31

**Authors:** Bernd Schneider, Ralf Kätzel, Michael Kube

**Affiliations:** 1 Thuenen-Institute of Forest Genetics, Eberswalder Chaussee 3A, 15377 Waldsieversdorf, Germany; 2 Landeskompetenzzentrum Forst Eberswalde, Alfred-Möller-Straße 1, 16225 Eberswalde, Germany; 3 Department of Integrative Infection Biology Crops-Livestock, University of Hohenheim, Garbenstr. 30, 70599 Stuttgart, Germany

**Keywords:** Phytoplasmas, Nationwide screening, TaqMan assay, Tolerance

## Abstract

**Background:**

**‘***Candidatus* Phytoplasma ulmi’ is the agent associated with elm yellows and has been categorised in the European Union as a quarantine pathogen. For central and northern European countries, information on the occurrence and distribution of the pathogen and its impact on elms is scarce, so a survey of native elm trees has been conducted in Germany.

**Results:**

About 6500 samples from *Ulmus minor*, *Ulmus laevis* and *Ulmus glabra*, were collected nationwide. Phytoplasma detection was performed by applying a universal 16Sr DNA-based quantitative PCR (qPCR) assay and a novel ‘Ca. *P. ulmi*’ specific qPCR assay targeting the 16S–23S spacer region. Both assays revealed that 28% of the samples were infected by ‘*Ca. P. ulmi*’, but infection rates of the elm species and regional incidences differed. The phytoplasma presence in the trees was not correlated to disease-specific symptoms. The survey identified a regional disparity of infection which was high in east, south and central Germany, whereas only a few infected sites were found in the western and northern parts of the country. Monitoring the seasonal titre of *‘Ca. P. ulmi*’ in an infected tree by qPCR revealed a high colonisation in all parts of the tree throughout the year.

**Conclusions:**

‘*Ca. P. ulmi*’ is widely present in elms in Germany. The rare occurrence of symptoms indicates either a high degree of tolerance in elm populations or a low virulence of pathogen strains enabling high infection rates in a long-living host.

## Background

Phytoplasmas are obligate parasites from the bacterial class Mollicutes, where they form the monophylogenetic taxon ‘*Candidatus* Phytoplasma’ [1]. They colonise the nutrient-rich phloem sap of their plant host and rely for transmission on phloem-feeding hemipteran insect vectors [2].

Phytoplasmas are associated with diseases of more than 1000 plant species, including many important crops [3]. Several phytoplasma diseases comprise the description ‘yellows’ or ‘yellowing’ in their name, due to the associated presence of leaf chlorosis. This is also the case for ‘*Candidatus* Phytoplasma ulmi’ infecting elm trees [4]. It is phylogenetically closely related to economically important plant pathogenic phytoplasmas such as “flavescence dorée” [5], ‘*Ca.* Phytoplasma rubi’ associated with rubus stunt [6], ‘*Ca.* Phytoplasma ziziphi’ associated with jujube witches’ broom [7], and alder yellows phytoplasma, associated with alder yellows [8]. These phytoplasmas belong to the elm yellows group and are classified as 16SrV group members based on their restriction fragment length polymorphism pattern in PCR-amplified 16Sr DNA [4].

Elm yellows was first described in 1938 in North America [9], but historic reports indicate earlier sightings [10, 11]. Most North American elm species are highly susceptible to an infection by the bacterium and show a dramatic course of disease progression [12–14]. The trees usually die within two years post-infection, displaying a number of characteristic symptoms such as leaf yellowing, witches’ broom formation and phloem necrosis. In the US, the disease spread gradually from the mid-western states to the east and to the south, causing a considerable loss of native elm trees [14]. In Europe, the disease was first reported in Italy and then later on in France, Bulgaria, Serbia and Croatia [15–17]. The disease symptoms displayed by the European elm species resembled those of the North American elm species, but phloem necrosis did not occur. Therefore, the European elm species were considered less susceptible than their American relatives [18].

Since 1975, ‘*Ca. P. ulmi*’ has been regarded in the EU as a harmful organism and regulated by the Council Directive 2000/29/EC [19]. A comprehensive analysis on the re-categorisation of ‘Ca. *P. ulmi*’ was conducted by the European Food and Safety Authority in 2014 [20], but due to limited information on its distribution, strain virulence, potential insect vectors and effects on European elm species, the report remained inconclusive. Ensuing reports of elm yellows findings from the UK, the Czech Republic, Poland and Belgium, however, demonstrated that ‘Ca. *P. ulmi*’ is more widespread in the EU than previously thought [21–24]. In response to the new situation the European Plant Protection Organization moved ‘Ca. *P. ulmi*’ in 2017 from Annex I list A1, as a pathogen absent from the EU, to list A2 [25]. In December 2019, the Council Directive 2016/2031 has deprived the quarantine status of ‘Ca. *P. ulmi*’ for continental Europe, but the status for Great Britain remains in place [26].

In Germany, ‘*Ca. P. ulmi*’ was first reported in 1992 from a single Scots elm (*Ulmus glabra*) displaying witches’ broom symptoms in south-western Germany [27]. DNA-DNA hybridisation studies with elm yellows-specific probes resulted in identical hybridisation profiles between German, French, Italian and American accessions, thereby providing evidence that the European elm yellows strains were closely related to the American strains [16]. In a more recent study, 59 European white elm (*Ulmus laevis*) trees, four of which showed stunted growth and leaf chlorosis, were examined in the states of Brandenburg and Berlin [28]. Based on 16Sr DNA nested PCR assays, half of the tree samples were identified as phytoplasma-positive, and sequence analyses confirmed the presence of ‘*Ca. P. ulmi*’. However, all symptomatic elm trees tested phytoplasma-negative. The unexpected high presence of ‘*Ca. P. ulmi*’, and the absence of disease symptoms found in this study, prompted a nationwide survey of the pathogen’s distribution and occurrence in the native elm species *U. glabra*, *U. laevis* and *U. minor*. The results of this survey, comprising a distribution map with incidence levels at sampling sites and a newly designed ‘*Ca. P. ulmi*’-specific qPCR assay, are presented herein.

## Results

### Wide absence of yellows disease symptoms in elm in Germany

A total of 6486 elm samples from 339 sites were collected. The plant samples comprised 2630 Scots elm, 204 8 European white elm and 180 8 field elm samples (Table 1**,** Fig. 3a). The individuals’ ages ranged from one-year-old seedlings to trees of more than 400 years. The trunk diameter of the trees ranged from 0.5 cm to 3.5 m.

**Table 1:** Summary of elm samples collected during the survey in Germany

Federal state and relative geographic position in Germany	Elm species and no. of samples			Total no. per state
	*U. glabra*	*U. laevis*	*U. minor*	
Baden-Württemberg (south-west)	371	90	265	726
Bavaria (south-east)	374	90	124	588
Berlin (east)	0	34	46	80
Brandenburg (east)	204	412	304	920
Hamburg (north)	0	0	29	29
Hesse (central)	70	15	25	110
Lower Saxony (north)	113	59	102	274
Mecklenburg West-Pomerania (north-east)	467	494	40	1001
Rhineland-Palatinate (south-west)	160	91	129	380
North Rhine-Westphalia (west)	223	228	231	682
Saarland (west)	29	0	31	60
Saxony (south-east)	270	180	150	600
Saxony-Anhalt (east)	46	138	170	354
Schleswig-Holstein (north)	215	142	78	435
Thuringia (south-east)	88	75	84	247
Number of species and total number	2630	2048	1808	6486

**Fig. 1:**
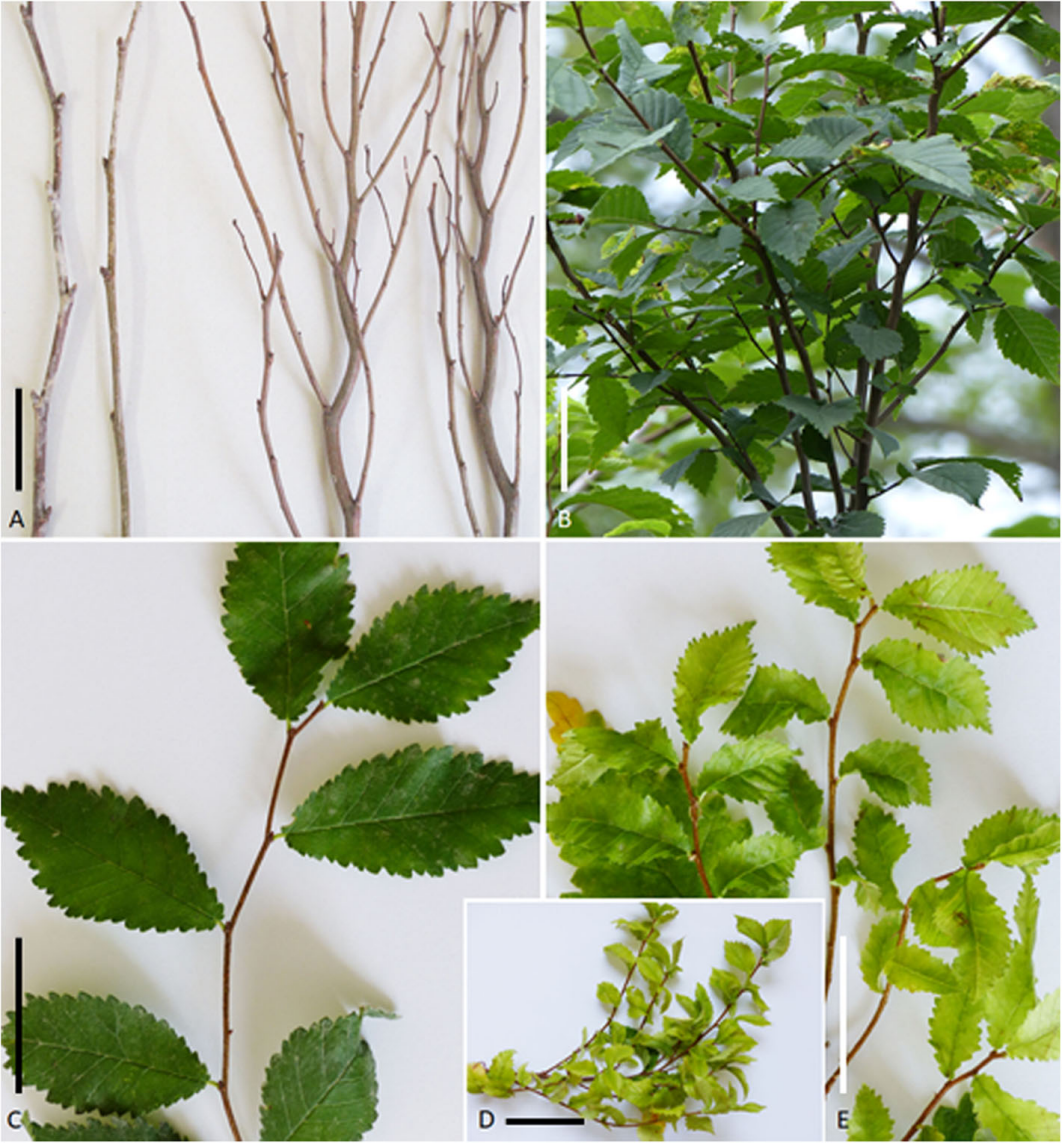
‘*Ca.* P. ulmi’-specific disease symptoms. **a** Branches of a healthy (left) and diseased (right) *U. glabra* tree in winter time. **b** Rare witches’ broom formation of *U. glabra* in summer time. For an overview of trees see Schneider and Kube, 2019 [29]. **c** Twig of a healthy *U. minor* with normal leaf size and leaf colouration showing mottle similar to virus infection. **d***U. minor* branch with stunted growth. **e***U. minor* with little leaf symptom and leaf chlorosis. The scale bar represents a length of 8 cm

‘*Ca. P. ulmi*’-specific symptoms were rarely observed, considering the number of trees, the infection rate at some sites, their different ages and environment. However, in 2017, 2018 and 2019, more than 15 symptomatic Scots elms, approximately 2 to 3 m in height, were observed along roadsides in Müncheberg, Brandenburg (Fig. 1a, b). The trees showed numerous witches’ brooms clearly visible in winter time and during new shoot development in July/August. These plants showed an early bud break at the witches’ broom sites in March compared to non-symptomatic parts of the trees. The branches and leaves outside the witches’ brooms resembled those of healthy trees. One tree, about the same height and in the same area, showed all branches severely stunted, and the leaves small and brittle. In 2018, field elms displaying little leaf, yellowing and stunting symptoms were observed in Ingelheim am Rhein, Rhineland-Palatinate (Fig. 1c, d, e) and near Haßfurt, Bavaria. In many natural habitats of Scots elms and field elms, the assessment on the presence of elm yellows symptoms was severely compromised by the Dutch elm disease which caused wilting and dieback. These symptoms were aggravated during the 2018 summer drought in Germany. Despite the fungal infection, samples were included in the survey.

### ‘*Ca. P. ulmi*’-specific TaqMan assay

**Fig. 2:**
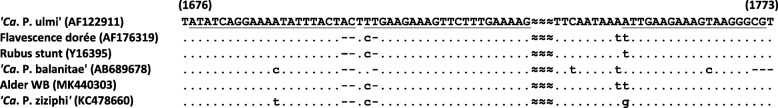
Alignment of 16S–23S spacer regions (5′ – 3′) of ‘*Ca.* P. ulmi’ and closely related phytoplasma strains. Nucleotide differences of the other sequences to the ‘Ca. P. ulmi’ sequence are indicated by lowercase letters. The positions of the forward primer, the TaqMan probe and the reverse primer are underlined and given from left to right, respectively. -, indicates gaps in the alignment. ≈, indicates bases not depicted. Numbers above the alignment correspond to nucleotide positions relative to the ‘Ca. P. ulmi’ sequence deposited under acc. no. AF122911

A universal quantitative real-time assay for the detection of phytoplasma presence and for control of the template quality (18Sr DNA plant) was routinely used [30]. A specific-assay for the detection of ‘*Ca. P. ulmi*’ was developed in this work. The universal and the specific assay have been applied within this study enabling the detection of phytoplasma presence and of ‘Ca. *P. ulmi*’ in particular, respectively. The selected forward and reverse primers and the TaqMan probe of the specific assay are located in the spacer region at positions 1677 to 1698, 1700 to 1721 and 1754 to 1772, respectively, relative to the first nucleotide of the ‘Ca. *P. ulmi*’ sequence deposited in GenBank under the accession number AF122911 and amplified a fragment of 96 bp. The ‘Ca. *P. ulmi*’ sequences showed the majority of the differences to the sequences of alder witches’ broom, “flavescence dorée”, rubus stunt, ‘*Ca*. P. balanitae’ and ‘*Ca*. *P. ziziphi*’ in the region between the forward primer (fEY_spacer-rt) and the TaqMan probe (qEY_spacer-rt) annealing sites. The reverse primer (rEY_spacer-rt) differs only by a T or a G at the 3′ end relative to the sequences of alder witches’ broom, “flavescence dorée”, rubus stunt and ‘*Ca*. *P. ziziphi*’, resepectively, or by an internal nucleotide difference relative to ‘Ca. P. balanitae’ (Fig. 2). Temperature gradient assays revealed an optimum temperature of 56 °C, for both product yield and assay specificity. At this temperature, only the ‘Ca. *P. ulmi*’ strains ULW and EYC maintained in *Catharanthus roseus* and the ‘*Ca. P. ulmi*’-infected field samples were amplified (data not shown).

To assess the number of phytoplasmas in the phloem tissue of elm samples, DNA standards consisting of cloned 16S–23S ribosomal DNA from strain ULW with copy numbers from 10^08^ to 10^01^ per μl were PCR-amplified with the universal phytoplasma and ‘*Ca. P. ulmi*’-specific assays. One μl of DNA extract from a healthy elm tree was added to each reaction, to simulate the assay conditions with unknown samples. Both assays showed a dynamic range of amplification with Ct values of 18.2 to 37.6 for the lowest and highest dilution, respectively, with serial dilution steps differing by Ct values of 2 to 3 (Table 2).

**Table 2:** Ct values of DNA standards after qPCR with the universal phytoplasma- and ‘*Ca. P. ulmi*’-specific spacer qPCR assay

Copy number/assay	Universal phytoplasma assay^a^	‘Ca. *P. ulmi*’-specific spacer assay^a^
10^08^	19.7	21.6
10^07^	21.4	23.2
10^06^	22.5	24.3
10^05^	25.7	27.1
10^04^	30.0	30.4
10^03^	31.3	32.0
10^02^	34.5	35.4
10^01^	37.2	35.1

^a^, Mean of four technical replicates including DNA from a non-infected elm tree

### Quantitative real-time PCR results highlight high incidence of infection in elm stands

The internal 18Sr DNA amplification control showed strong amplification (Ct values 9 to 22), due to the high content of plant DNA but also confirmed the template quality (data not shown). Samples with a Ct value > 22 were re-assayed, or the DNA extraction was repeated. With the universal phytoplasma assay, 1803 of the 6486 elm samples were rated phytoplasma-positive, representing an infection rate of 27.8% based on the total number of samples (Table 3). With the specific qPCR assay 1801 samples tested positive (Table 3). To estimate the number of phytoplasmas present in the positive samples, four Ct categories were established. With the universal qPCR assay the majority of the positive samples grouped in the Ct range  > 22 ≤ 28 followed by the Ct range > 18 ≤ 22 representing 80% of all positive samples. Almost 7% of the positive samples revealed Ct values below 18. The specific qPCR assay had a different performance. Here, the majority of positive samples grouped in the Ct rage > 22 ≤ 28, followed by the Ct range > 28 ≤ 34. In comparison with the universal qPCR assay, 73% of positive samples grouped between Ct 18 and Ct 28. Only 5 samples showed Ct values of 18 or below. However, considering the set threshold level of Ct ≤ 34 both assays identified the same number of ‘*Ca. P. ulmi*’-positive samples.

**Table 3:** Number of samples listed by Ct categories obtained with the universal phytoplasma- and specific qPCR assays

qPCR Assays and elm species	Ct Category ^a^				Sum of rows
	> 28 ≤ 34	> 22 ≤ 28	> 18 ≤ 22	≤ 18	
Universal phytoplasma assay					
*U. glabra*	66	314	271	100	751
*U. laevis*	103	465	90	7	665
*U. minor*	74	196	103	14	387
Sum of columns	243	975	464	121	1803
‘Ca. P. ulmi’-specific assay					
*U. glabra*	105	514	127	4	750
*U. laevis*	240	412	11	1	664
*U. minor*	130	235	22	–	387
Sum of columns	474	1161	160	5	1801

^a,^ Columns refer to Ct categories and the associated number of positive samples

Scots elm trees showed in general a higher phytoplasma titre compared to the other elm species. However, there was no correlation between the phytoplasma titre and ‘*Ca. P. ulmi*’-symptoms (data not shown). The amplification curves in assays with the DNA of a non-infected elm tree and a no-template control always ranged below the threshold line.

### Comparison of qPCRs revealed rare occurrence of other phytoplasmas

The ‘*Ca. P. ulmi*’-specific assay detected two positive samples less than the universal phytoplasma assay (Table 3). The difference in the two cases was due to trees infected by other phytoplasma strains. Partial sequence analysis of two P1/P7 PCR fragments revealed that one phytoplasma sequence (1631 bp) was identical to the sequence of “flavescence dorée” phytoplasma strain FD70 (acc. no. AF176319) from France, whilst the other sequence (1631 bp) was identical to phytoplasmas found in *Artemisia vulgaris* (acc. no. MK440304) and *Alnus glutinosa* (acc. no. MK440303) in Poland. An alignment of both sequences to the ‘*Ca. P. ulmi*’ sequence AF122911 revealed six nucleotide exchanges within the 16S gene and mispairing at the binding sites of primers and probe of the specific assay. However, the 16Sr RNA gene of both phytoplasmas was identical to “flavescence dorée” phytoplasma strain FD70 which is a member of the elm yellows group 16SrV-C. The sequences have been deposited in GenBank under the accession numbers MN394841 and MN394842.

### Distribution map of ‘*Ca. P. ulmi*’ in Germany highlights the presence of hot spots

**Fig. 3:**
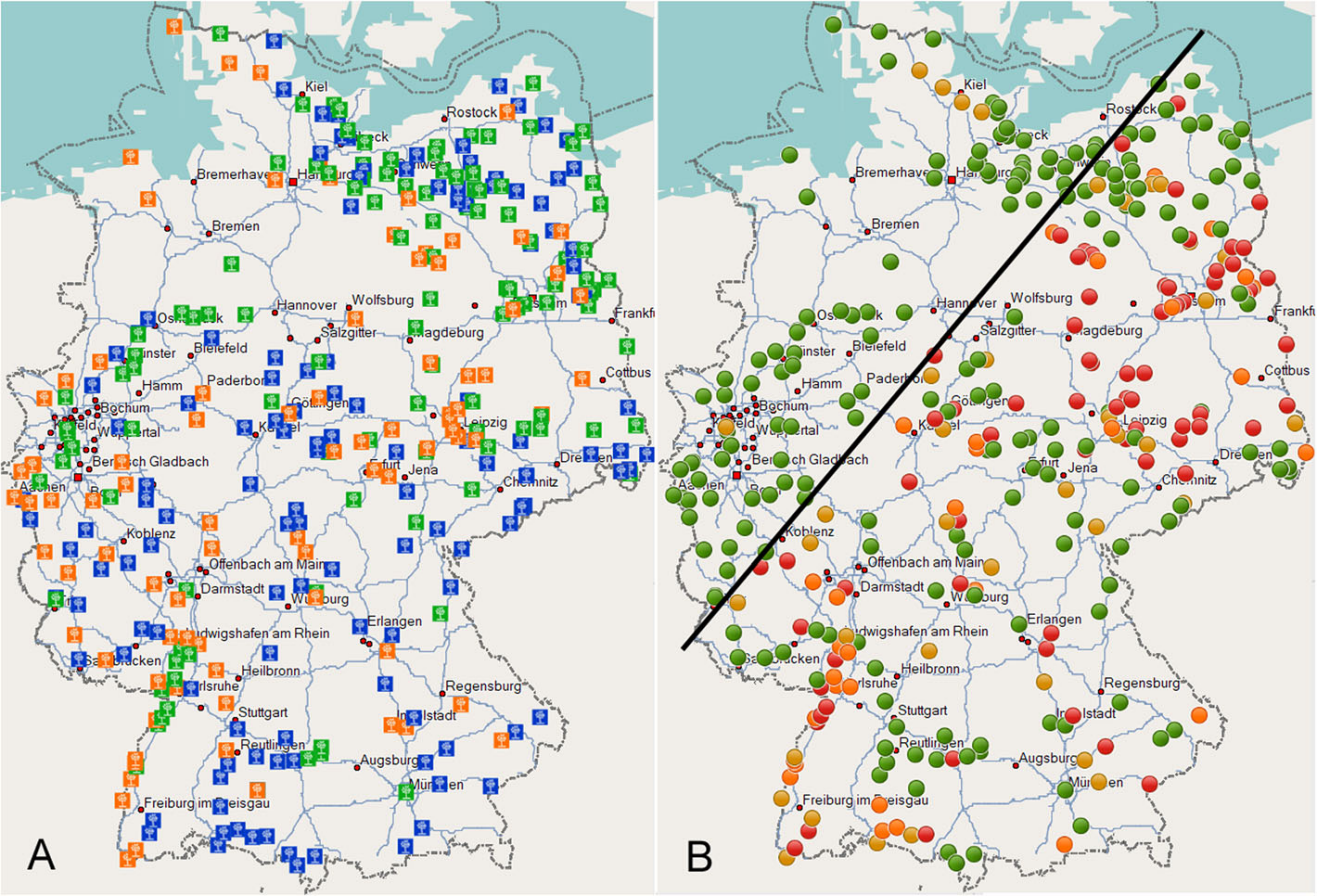
**a**, sampling sites with predominant elm species. Blue square, *U. glabra*; Green square, *U. laevis*; Orange square, *U. minor*. **b**, presence of ‘*Ca.* P. ulmi’ at the sampling sites based on qPCR results. Green dot, no phytoplasmas found; Yellow dot, up to one-third of trees infected; Orange dot, more than one-third and up to two-thirds of trees infected; Red dot, more than two-thirds and up to 100% of trees infected

Elm samples were collected at 339 sites in Germany. The elm species were not homogeneously distributed across the territory (Fig. 3a). The approximate species frequency in the federal states is reflected by the numbers given in Table 1. The detected presence of ‘*Ca. P. ulmi*’ was likewise not homogeneous (Fig. 3b). Regions with sites showing an infection rate of more than 66.7% were clustered in Saxony, Saxony-Anhalt and Brandenburg. Other hotspots occurred along the upper Rhine valley, and some were present in Bavaria and Hesse. Sites showing a lower infection rate were mostly found in the vicinity of these hotspots. The infection rate decreased towards the west and north, and only five sites were found above a virtual line drawn from Trier to Rostock (Fig. 3b). At one of these sites in North Rhine-Westphalia, *U. laevis* showed an infection rate of 30% (six out of 20 samples). The four sites in Schleswig-Holstein showed infection rates of 2.5% (one out of 40 samples) and 20% (four out of 20 samples) for *U. glabra*, and 5% (one out of 20 samples) and 15% (six out of 40 samples) for *U. laevis* and *U. minor*, respectively.

### Infection rate correlated to altitude or tree age

**Fig. 4:**
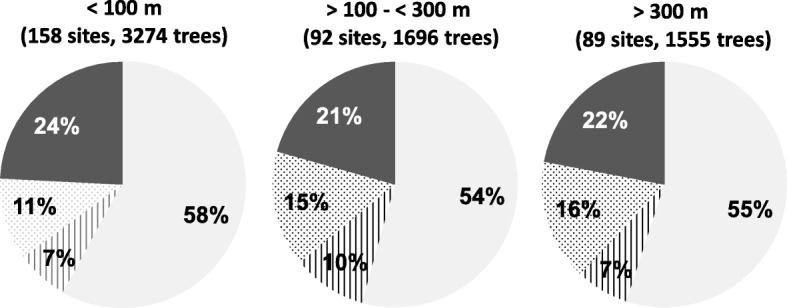
Overall infection rate of elm species at different altitude levels. Altitude, number of sites and number of individuals examined indicated. Left (light grey), percentage of sites with non-infected trees. In a clockwise direction, percentage of sites with up to one-third, more than one-third and up to two-thirds and more than two-thirds of individuals infected by ‘*Ca.* P. ulmi’

The majority of sampling sites were located in the German lowlands at altitudes ≤100 m above the average mean sea level (AMSL). However, quite a few sites were also located in the low mountain range, from 300 to 1100 m AMSL. About the same number of sites ranged in between (Fig. 4). The proportion of sites free of phytoplasmas, and those with a low (up to 1/3 of individuals), high (up to 2/3 of individuals) and extreme infection rate (up to 100%), were almost identical among the zones, thereby indicating a broad habitat for potential insect vectors.

**Fig. 5:**
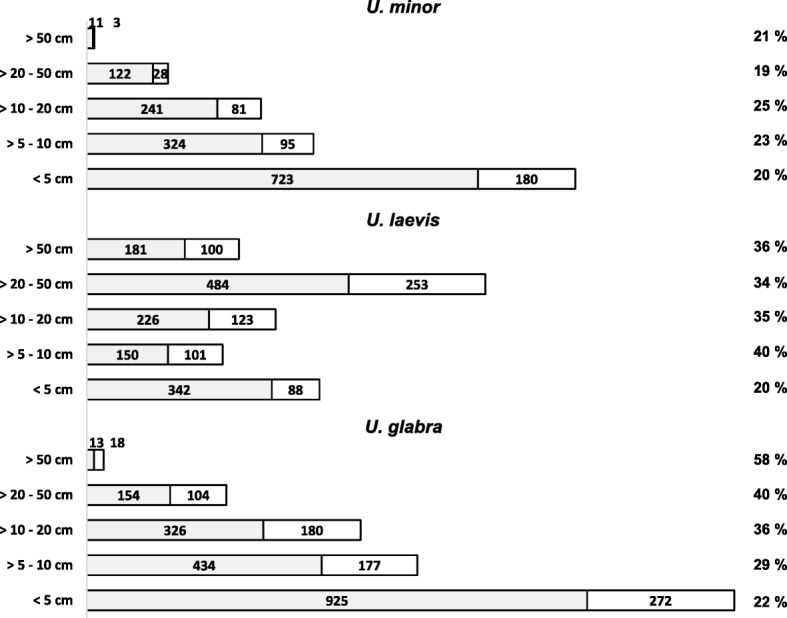
Infection rate of *U. minor*, *U. laevis* and *U. glabra* in relation to trunk diameter. Grey bars represent non-infected individuals. White bars represent phytoplasma-infected individuals. The number of individuals considered is given in the bars. The infection percentage in an age group is indicated on the right

Except for monumental trees and seedlings, the ages of trees were unknown and calculated on the basis of the trunk diameter. For trees up to 5 cm, 10 cm, 20 cm and 50 cm in diameter, age was calculated at 10, 20, 39 and 98 years, respectively. The oldest tree was a European white elm in Gülitz (Brandenburg), estimated to be 400 to 700 years old and with a diameter of 3.5 m. The number of infected individuals was determined in relation to trunk diameter (Fig. 5). The graph revealed different disease progressions for the three species. While *U. glabra* showed a steady increase of infection with age, *U. laevis* showed a strong increase of infected individuals in trees up to 20 years of age, reaching a plateau thereafter. A different situation was observed for *U. minor*. An infection rate of 20% was determined in the youngest age group, and this number did not change much in the other age categories. The graph also shows the diminishing number of older trees for Scots elms and field elms, due to the mortal effects of Dutch elm disease on their population. Of particular interest was the situation of old and monumental trees of more than 100 cm in trunk diameter. 111 trees with an estimated age of 195 years and older were included in this survey. Thirty-eight individuals were infected by ‘*Ca. P. ulmi*’, comprising 29 *U. laevis-,* eight *U. glabra-* and one *U. minor* tree.

### All-season colonisation of elm

To assess the seasonal fluctuation of pathogen numbers, a monthly screening of different plant parts from an infected Scots elm tree was performed with the universal qPCR assay. The mean monthly Ct values of all samples within the examined period ranged from 24.2 to 28.6. The June to December Ct means were slightly lower than the January to May means. The Ct means between trunk samples and root samples of the same month never differed by more than 3. The Ct values of the individual monthly trunk samples were close and never apart by more than four cycles. The lowest phytoplasma titre was recorded from January to March in buds, with Ct means ranging from 28.4 to 31.5. Considering the lowest average Ct (24.2) value, a phytoplasma number of 10^08^ per gram of phloem tissue was calculated. The highest Ct average (Ct 31.5) found in bud material represents a phytoplasma number two orders of magnitude lower.

## Discussion

A recent survey in the states of Brandenburg and Berlin identified an unexpected high infection rate of *U. laevis* by ‘*Ca. P. ulmi*’ [28]. To verify the situation on a nationwide scale a representative survey with a high number of samples has been conducted covering most of the natural habitats of the three elm species. Preliminary results of this survey have been published [29].

At the beginning of the survey, no information was available on the colonisation of ‘*Ca. P. ulmi*’ in infected elm trees. In the well-studied phytoplasma diseases of pome fruit trees, the phytoplasma titre displays strong seasonal fluctuations, reaching a low in late winter to early summer [31, 32]. To obtain a first insight on the colonisation pattern of an elm tree by ‘Ca. *P. ulmi*’ a naturally infected *U. glabra* tree was taken as study object. The analysis revealed that ‘Ca. *P. ulmi*’ colonises roots and shoots all year round in a constantly high titre. Therefore, branches or trunk material were sampled throughout the survey. Even though petioles or leaf midribs would have been easier to collect, the choice fell on branches or trunk material, as the sampling period could be extended with this material. However, a more extended monitoring of infected elm trees is necessary to generalise these preliminary colonisation behaviour results also for infected *U. laevis* and *U. minor* trees.

The qPCR assays corroborated the results of the seasonal course study, as in most trees a fairly high number of ‘*Ca. P. ulmi*’ was identified, exemplified by a Ct value ≤28 representing an organism titre of 10^06^ per gram of phloem tissue and higher. Both qPCR assays worked reliably, although the specific assay showed a slightly lower performance in respect to the Ct values. This was most likely caused by the lower G + C content of the primers and probe and the reduced binding strength compared to the oligonucleotides of the universal phytoplasma assay. The substitution of adenine bases with 2,6-diaminopurin, to increase the melting temperature of the TaqMan spacer probe, did not change the performance significantly compared to the assay with the non-modified probe. Even though, in comparison with the universal qPCR assay, the spacer assay proved to be a reliable diagnostic tool in terms of positive calls for the presence of ‘*Ca. P. ulmi*’.

It is evident that ‘*Ca. P. ulmi*’ was present in almost 28% of the elm samples, thus indicating the high number of infected trees in the German elm populations. A higher overall ‘Ca. *P. ulmi*’ infection rate of 46% was found in Croatian elm populations [33]. Those and higher numbers were also observed in Germany if the evaluation would had focused on phytoplasma hotspots only. The high overall incidence rate in Croatia is therefore a matter of sample size. Other studies reported of infection rates as high as 85%, but this is caused by a preferential collection of material from symptomatic trees which does not reflect the real infection rate [34]. The three elm species showed different disease rates. While almost one-third of the *U. laevis* samples tested ‘*Ca. P. ulmi*’-positive, *U. glabra* and *U. minor* were infected to a lesser degree. This difference is not due to sample size, as the number of tested trees from each species was about similar. In Croatia, elm species were also infected to a different degree, in which almost 75% of *U. laevis* trees were infected, followed by *U. minor* with 10.8% and *U. glabra* with 4% [33]. However, in this work, the latter two species were also infected by other phytoplasmas, and therefore the infection rates are not directly comparable.

In all, except for two elm samples, ‘*Ca. P. ulmi*’ was identified. These phytoplasmas were detected by the different specificity of the two qPCR assays employed. Sequence analyses of ribosomal fragments revealed that both phytoplasmas were members of the 16SrV, or elm yellows group. But in contrast to ‘Ca. *P. ulmi*’ which taxonomically group in subgroup A, both isolates belonged to subgroup C. ‘Ca. Phytoplasma solani’ and ‘Ca. Phytoplasma asteris’, have been described in *U. glabra* and *U. minor* displaying rather unspecific symptoms of leaf yellowing and drying [33]. These symptoms could not be observed in the two phytoplasma-infected trees. The presence of other phytoplasmas in elms is most likely due to an occasional feeding of infected insect vectors. However, the fact that only 16SrV-group phytoplasmas were found in elm trees might indicate a host-pathogen specificity at the plant host- or insect vector level, or on both.

Despite the high number of phytoplasmas in the sieve tubes, German elms seem to react in a tolerant way upon infection, which stands in striking contrast to the reactions of American and Asian elm species [14, 18]. The few symptomatic *U. glabra* and *U. minor* trees that were found, however, displayed typical ‘*Ca. P. ulmi*’ symptoms with witches’ brooms, stunting and leaf chlorosis. Mittempergher (2000) also concluded after extended observations in Italian elm breeding stations that European elm species tolerate a ‘Ca. *P. ulmi*’ infection quite well, although the number of symptom sightings from southern Europe [33–35] for *U. minor* and *U. laevis* seem to be more frequent compared to reports from central and northern regions of the continent. However, this could also be linked to regional differences of ‘Ca. *P. ulmi*’ strain virulence, climatic factors, genetic background of elm populations or other undetermined stress factors.

The different infection rates of the elm species and within the age categories are difficult to explain. The progressive infection in aging *U. glabra* populations is easily comprehensible, but the plateau phase for *U. laevis*, and the constant infection rate of *U. minor* age classes, is not as easy to deduce. The same is true for the old and monumental trees. How some of these trees escaped phytoplasma infection remains unclear, as they were well located in regions of high infection pressure. Therefore, it seems that a certain degree of resistance is present in the populations. However, other factors might be involved too, like recovery [36] or the protective role of the plants’ microbiome [37], albeit insufficient information is available to assess their influence so far.

Infection hotspots were located in the eastern, central and south-western parts of Germany, whereas infected sites became rare towards the north and north-western regions. The most plausible explanation is that an insect vector migrated from the southern-to-eastern side into the territory moving towards the north and west. Beside the verified ‘*Ca. P. ulmi*’ vector *Macropsis mendax* for Italy [38], phytoplasmas of the elm yellows group have been identified by PCR and PCR-RFLP analysis in *Hyalesthes luteipes* in Serbia, in *Iassus scutellaris*, *Allygidius furcatus* and *Cixus sp*. in France, but no transmission experiments were performed [39, 40]. For Germany, no data are available. The fact that infected trees were found from sea level up to an altitude of 750 m might indicate a vector different to *M. mendax*, as this insect is only known to occur up to an altitude of 400 m [41]. A spread of the bacterium through the exchange or trade of infected elms for planting purposes can be excluded, as elm timber has little forest use. In addition, root bridges can be discounted, as this would only explain spread in a small area.

This survey demonstrated a nationwide distribution of ‘*Ca. P. ulmi*’ in all three native elm species. The number of infected individuals is such that the eradication of this pathogen is impossible. The recent finding of ‘Ca. *P. ulmi*’ in Belgium [24] demonstrates its presence also west of Germany, although the confirmed number of infected elms is still low.

This work has established a sound basis for future research with respect to transmission and phytoplasma-host interaction. Examples of tolerance are often overlooked by phytopathologists and are rarely reported. It is remarkable that the closely related elm yellows and alder yellows phytoplasmas share this feature [42], which might be the result of a long-term co-evolution of these phytoplasmas with their hosts. A deeper understanding of such a particular phytoplasma-host interaction may provide the key to developing new strategies to cope with phytoplasma associated diseases in agriculture and forestry.

## Conclusions

This work presents the first nationwide survey of ‘*Ca. P. ulmi*’ presence in native elm species in Germany providing representative figures of infection incidence in the federal states. Almost 28% of all elm accessions tested ‘*Ca. P. ulmi*’-positive. Elm species were infected to a different degree with regional disparity of infection. Hot-spots of infection were identified in East-, Southeast and Central Germany while infection rates in West- and North Germany were low. Despite the high infection rate, disease symptoms were rarely found indicating a high degree of tolerance of native elm species to infection. An infection of elm trees by other phytoplasmas was only detected in two cases. A specific qPCR TaqMan assay based on 16S–23S spacer sequence motives has been developed providing sensitive and reliable detection of the pathogen. The occurrence of infected elm trees in regions beyond 400 m of altitude suggests insect vectors different to the verified ‘Ca. *P. ulmi*’-vector *M. mendax.* The American vector species *Scaphoideus luteolus* and *Allygus atomarius* do not occur in Germany, except *Philaenus spumarius*, which is widely distributed but failed to vector ‘Ca. *P. ulmi*’ in transmission trials. Therefore, it is likely that other unknown vectors are involved in ‘Ca. *P. ulmi*’ transmission.

## Methods

This study aims to clarify the infection status of elms in Germany with respect to ‘*Ca. P. ulmi*’. Therefore, the survey comprises the sampling of plant material from elms in Germany, followed by DNA extraction providing the templates for screening by quantitative PCR assays. Diagnostic qPCR assays were performed by applying universal phytoplasma primers [30] and a new specific primer set for ‘*Ca. P. ulmi*’ (this study).

### Sampling of plant material from elms

Elm samples were collected at 339 sites, based on a survey published in 2007 on the genetic resources of elm species in Germany [43]. The monumental elm tree sites were taken from the web resource on monumental trees (https://www.monumentaltrees.com). In addition, one- to two-year-old seedlings were obtained from nurseries in Riedlingen (Baden-Württemberg), Müncheberg and Waldsieversdorf (both Brandenburg). Additional elm trees were randomly sampled during the surveys at roadsides or in public parks. The diameter of each trunk was measured at a height of 1.3 m, and the approximate age was estimated by software provided on the website www.baumportal.de [44]. Elm shoot samples were collected from October 2017 until May 2019. Where possible, two shoot samples, about 25 cm in length and 0.5 to 4 cm in diameter, were collected from 20 randomly selected trees per site. The phytoplasma strains ULW (elm yellows phytoplasma, subgroup 16SrV-A) and ALY (alder yellows phytoplasma, subgroup 16SrV-C) maintained in *Catharanthus roseus* were used as reference strains [14, 45]. The coordinates of all collected elm accessions were recorded in WGS84 format, using a portable GPS device. The seasonal colonisation of ‘*Ca. P. ulmi*’ was monitored for one year in an infected Scots elm 3 m in height and 6 cm in diameter. Samples were taken on a monthly basis from the roots, at trunk ground level and then at distances of 25 cm up to a height of 1.75 m. Phloem tissue at these sites was extracted from a square of 0.5 cm^2^. Midrib or bud samples from the top and bottom of the tree were also examined.

### DNA extraction

DNA from all elm shoot samples was extracted from 125 mg of phloem tissue using 3 ml of CTAB buffer according to a procedure described by Ahrens and Seemüller [46]. The tissue was homogenised with a steel ball homogeniser in plastic extraction bags (Bioreba, Grenzach). The nucleic acids pellet was resuspended in 200 μl of sterile water and stored at − 20 °C until use. Small-scale phloem DNA extractions (≤ 15 mg) were performed with a Beadruptor (Biolab, Bebendorf) in microfuge tubes containing ceramic beads and 250 μl of CTAB buffer. The nucleic acids pellet was resuspended in 50 μl of sterile water and stored as described above. DNA from other phytoplasma strains (‘*Candidatus* Phytoplasma asteris’ strain AAY [subgroup 16SrI-B]; ‘*Candidatus* Phytoplasma mali’ strain AT [subgroup 16SrX-A]; ‘*Candidatus* Phytoplasma cynodontis’ strain BGWL [subgroup 16SrXII]; ‘Ca. *P. ulmi*’ strain EYC [subgroup 16SrV-A]; Western X disease, Green Valley strain GVX [subgroup 16SrIII-A]; ‘*Candidatus* Phytoplasma aurantifolia’ strain WBDL [subgroup16SrII]; ‘*Candidatus* Phytoplasma australiense’ [subgroup 16SrXII-B]; ‘*Candidatus* Phytoplasma rubi’ strain RuS [subgroup 16SrV-E]) maintained in *C. roseus* and “flavescence dorée” strain FD70 (subgroup 16SrV-C), maintained in *Vicia faba*, was obtained from Kerstin Zikeli and Michael Maixner (Julius Kuehn-Institute, Institute for Plant Protection in Fruit Crops and Viticulture, Germany) and were used as positive or negative controls.

### Quantitative PCR standard

The 16S-23Sr DNA of the ‘*Ca. P. ulmi*’ strain ULW was amplified with P1/P7 primers [47, 48] as described previously [49]. The 1.8 kb PCR fragment was ligated into the cloning vector pGEM-T (Promega, Madison), and the insert was verified by sequencing with vector primers (M13, M13rev) and the universal phytoplasma primers fU5 and rU3 [46]. The recombinant plasmid was bulk-extracted, its quantity determined by Qubit fluorometric quantification (Invitrogen, Carlsbad) and serially diluted with sterile water to obtain plasmid concentrations ranging from 10^08^ to 10^01^ copies per μl (referred to as ‘DNA standard’). P1/P7 PCR fragments of phytoplasma field strains were sequenced as described above. Sequences differing from ‘Ca. *P. ulmi*’ database entries were deposited at GenBank. The standard was only used to roughly estimate the plant phytoplasma titre and was not included in routine screenings.

### Calculation of phytoplasma titres

The phytoplasma titre was determined by the Ct values of the above mentioned serially diluted DNA standard relative to the Ct value of the elm samples. The calculation considered the amount of plant tissue represented in the nucleic acids pellet, the volume of the reconstituted nucleic acids pellet and the copy number of the target gene. The calculated figures are approximate values.

### Design of ‘*Ca. P. ulmi*’-specific primers

The 16S–23S spacer sequences from ‘*Ca. P. ulmi*’ database entries with acc. Nos. AF122911, AF189214 and EU184021 and spacer regions of related phytoplasmas (“flavescence dorée” phytoplasma strain FD70, acc. no. AF176319; rubus stunt phytoplasma, acc. no. Y16395; ‘Ca. P. balanitae’, acc. no. AB689678; ‘Ca. *P. ziziphi*’, acc. no. KC478660 and alder witches’ broom phytoplasma, acc. no. MK440303) were aligned using Clustal X [50]. The primers and probe were selected based on regions of complete homology to ‘Ca. *P. ulmi*’ sequences and the maximal number of base differences or gaps to the related phytoplasmas. The derived primers and probe were as follows (5′ – 3′): Forward primer fEY_spacer-rt, ATATCAGGAAAATATTTACTAC; reverse primer rEY_spacer-rt, CGCCCTTACTTTCTTCAAT; TaqMan probe pEY_spacer-rt, FAM-TTGAAGAAAGTTCTTTGAAAAG-BHQ1. Double underlined nucleotides were replaced by 2,6-diaminopurin to increase the melting temperature.

### Diagnostic qPCR assays

A TaqMan real-time PCR assay for universal phytoplasma detection was performed [30] with the following modifications. The assay was performed in 10 μl reactions containing 1 μl of nucleic acids extract, 10 pmol of each forward and reverse primer and a probe (5′-FAM/BHQ1–3′) for phytoplasma detection, 3.3 pmol of each forward and reverse primer and a probe (5′-Cy5/BHQ3–3′) for plant 18Sr DNA amplification and 5 μl of 2 x primaQUANT mastermix (Steinbrenner, Wiesenbach). The reactions were cycled as described previously [26] in a qTower (Analytik Jena AG, Jena), except that the initial 50 °C and 95 °C steps were replaced by a three-minute denaturation step at 95 °C. The plant 18Sr DNA assay served as an internal amplification control to exclude the presence of inhibitory co-extracted compounds. The ‘*Ca. P. ulmi*’-specific spacer assay was performed in 10 μl reactions containing 10 pmol of each forward and reverse primer and probe, albeit without plant-specific primers and probe. The cycling parameters were as follows: One cycle for 3 min at 95 °C, 40 cycles at 95 °C for 15 s and 56 °C for 25 s. Data evaluation was done via the cycler software provided by the manufacturer. Due to the high number of samples, verification of real-time PCR results was performed on a step-by-step basis. Samples were re-examined if the Ct value between universal and specific assays differed by more than three, or if one of the assays was rated negative. In each run, a phytoplasma-positive field sample with a Ct value of 29, ULW DNA (positive control), DNA of a healthy elm and a water control (negative controls) were included, to verify run consistency. The Ct values were assessed stringently, and samples with Ct values > 34 were considered negative.

## Data Availability

All data generated or analysed during this study are included in this published article. Specific datasets on coordinates of elm stands analysed during the current study are available from the corresponding author on reasonable request.

## Abbreviations

EU: European Union
Ct: Cycle threshold
AMSL: Average mean sea level
IPWG: International Phytoplasmologist Working Group
EPPO: European Plant Protection Organisation
CTAB: Cetyltrimethylammoniumbromide
